# First person – Rita Serrano

**DOI:** 10.1242/dmm.049435

**Published:** 2022-03-08

## Abstract

First Person is a series of interviews with the first authors of a selection of papers published in Disease Models & Mechanisms, helping early-career researchers promote themselves alongside their papers. Rita Serrano is first author on ‘ Novel preclinical model for CDKL5 deficiency disorder’, published in DMM. Rita is a postdoctoral researcher in the lab of Prof. Robert Bryson-Richardson at Monash University, Melbourne, Australia. Her research interests include neuromuscular disease modelling in zebrafish and identification of translational therapies.


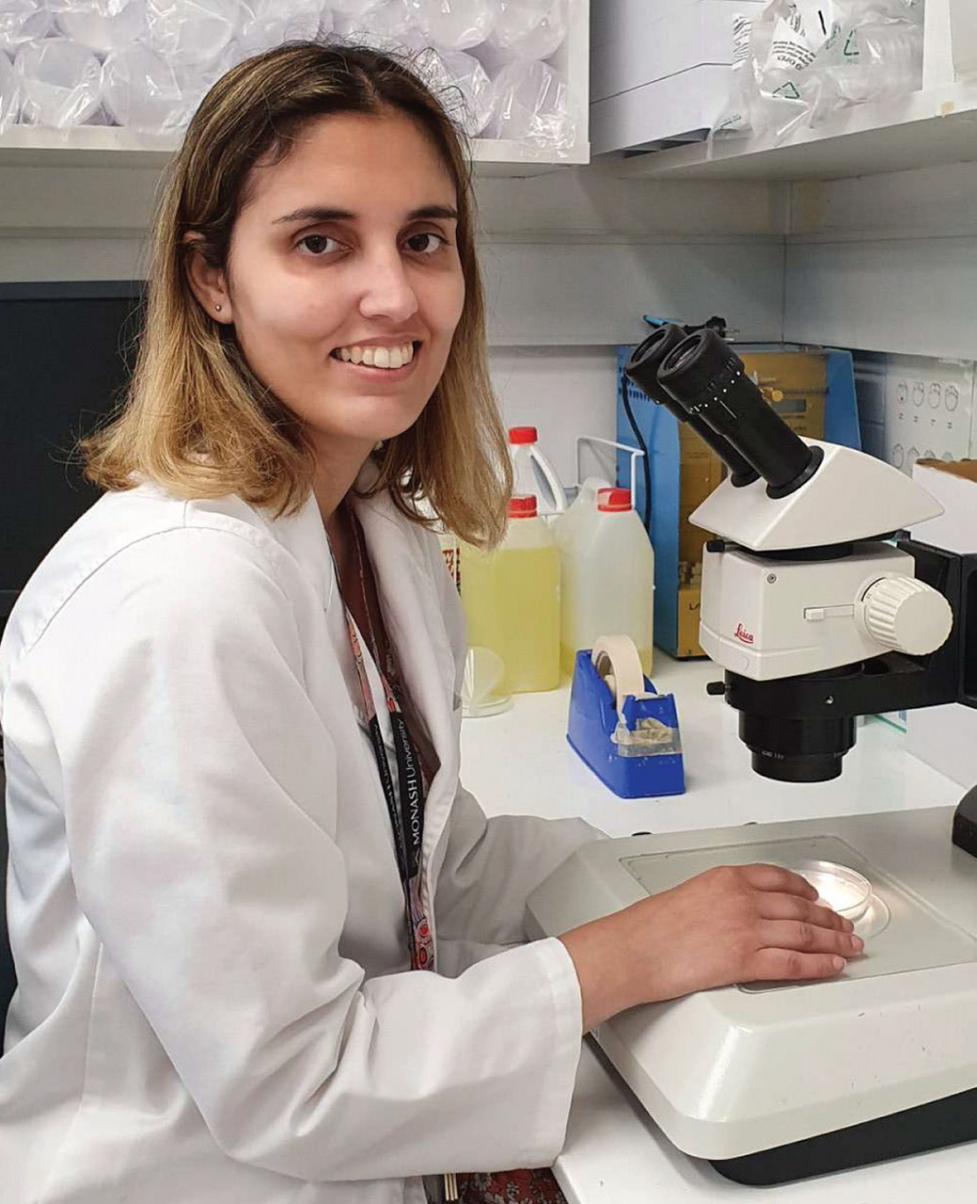


**Rita Serrano**

**How would you explain the main findings of your paper to non-scientific family and friends?**

CDKL5 deficiency disorder (CDD) is a severe neurological disorder caused by mutations in the *CDKL5* gene, and is characterized by early-onset seizures, intellectual disability and delayed motor function. We have described a zebrafish model for CDD that shows many of the symptoms observed in patients. We demonstrated that *cdkl5* mutant zebrafish display spontaneous seizure activity, reduced movement, reduced brain size and growth abnormalities. We also observed defects in nerve formation in *cdkl5* mutant zebrafish, which likely result in impaired neuronal communication, leading to seizures and reduced brain function. Our study validates the zebrafish model as a reliable system to explore CDD and identify potential drugs that can improve patients' quality of life. 

**What are the potential implications of these results for your field of research?**

CDD is a relatively understudied disease for which the molecular mechanisms that lead to neurodegeneration are unknown. Our zebrafish model will allow the detailed examination of disease pathogenesis and understand how the loss of CDKL5 function contributes to the symptoms observed in CDD patients. In addition, we provide a robust platform to test potential treatment options that can be directly translated to patients, in particular, drugs that can be tested in zebrafish in a large-scale and time-efficient manner.

**What are the main advantages and drawbacks of the model system you have used as it relates to the disease you are investigating?**

Zebrafish as a model has many advantages for the study of neurological disorders, like CDD. Many brain structures and signalling pathways are conserved between humans and zebrafish, which allows us to visualize different neurodegeneration stages and compare the findings with patients' clinical data. As zebrafish embryonic development is external, it is highly suitable for *in vivo* examination of neural development, and therefore neurodevelopmental disorders. Additionally, zebrafish as a model provides great statistical power for any type of study performed due to the high number of offspring that can be used in each experiment. One major drawback of using zebrafish is that many of the tools available, for example antibodies that are able to recognize zebrafish proteins, are not available, and therefore protein level cannot always be determined.

“The use of live imaging and transgenic strains to visualize brain signalling during epileptic bursts […] has considerably improved the analysis of epileptic seizures.”

**Figure:**
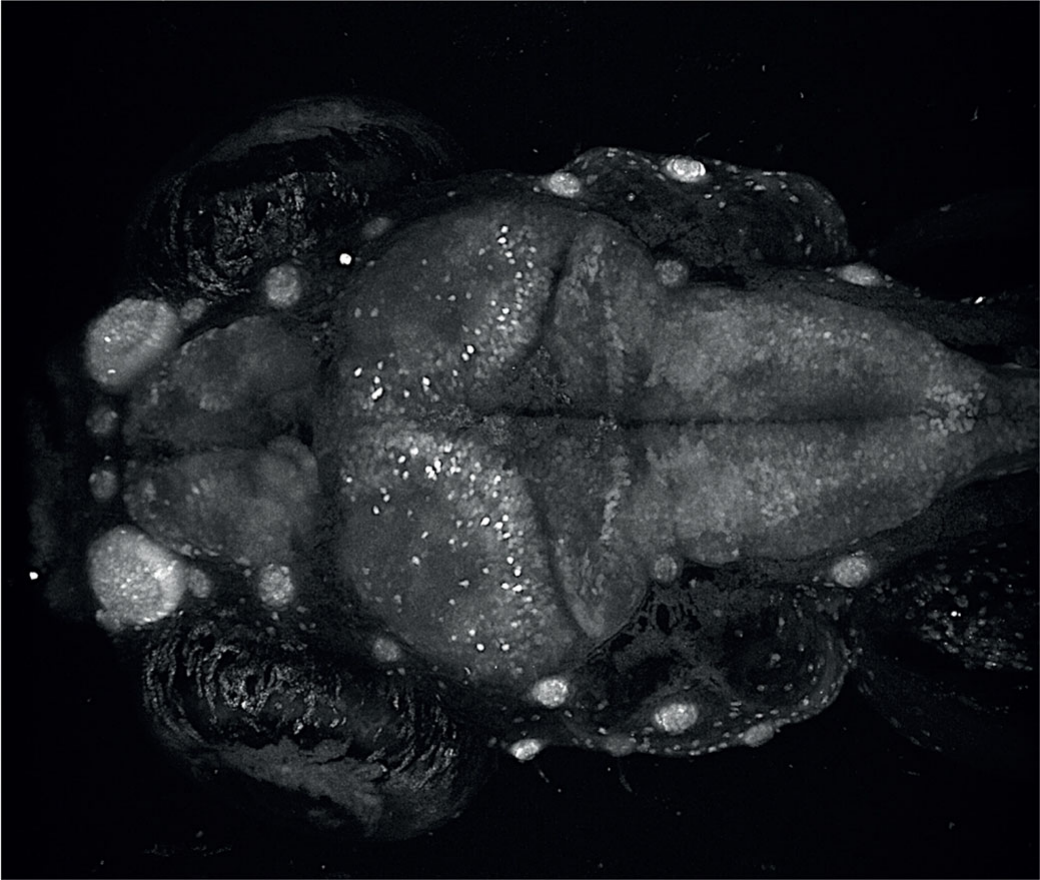
Maximum-intensity projection of a Tg(*HuC:EGFP*) zebrafish brain at 6 dpf.

**What has surprised you the most while conducting your research?**

The increasing development of molecular tools to examine brain morphology and activity in zebrafish is fascinating. Namely, the use of live imaging and transgenic strains to visualize brain signalling during epileptic bursts, which we have used in this work, has considerably improved the analysis of epileptic seizures compared to previous methodologies that are based on movement alone. Also, I am always impressed by how such a small vertebrate animal, like the zebrafish, can phenocopy many human disorders, in particular neurological disorders, which often exhibit a complex array of clinical symptoms.

**Describe what you think is the most significant challenge impacting your research at this time and how will this be addressed over the next 10 years?**

The most significant challenge impacting my research is the support dedicated to research into rare neurological diseases. Individually, these diseases affect a small part of the community; however, together, rare diseases impact a huge proportion of the population. Hopefully over the next 10 years, there will be more awareness of patients (and their families) impacted by rare neurological diseases and this will be translated into increased funding to develop robust disease models which can be used to identify effective treatments.

“It is not uncommon to hear about grant rejections and that there aren't sufficient funds to continue a promising project, making the prospects as a researcher gloomy.”

**What changes do you think could improve the professional lives of early-career scientists?**

I consider myself fortunate to have had an amazing supervisor that supported me during my PhD studies and now supports me as a young postdoctoral researcher. Others in my laboratory have also been instrumental in guiding my research investigations, including Dr Tamar Sztal, the research fellow driving this research. Currently, it is not uncommon to hear about grant rejections and that there aren't sufficient funds to continue a promising project, making the prospects as a researcher gloomy. Our society does not consider the postdoctoral career yet as a profession but rather as a transition period. The transition period, however, is often a more permanent stage due to limited faculty positions and insufficient funding to support researchers after years of commitment to their research, resulting in many researchers leaving this career path. I wish I could see the day that research is an important career and a long-term job for those who have the motivation and passion for it. At my current career level, however, I think short-term funding programmes to learn new technical skills could improve research quality and future employability.

**What's next for you?**

I am continuing my research with additional zebrafish models of epilepsy and neuromuscular disease in Australia. I am interested in unravelling the signalling pathways that trigger these, often rare and debilitating, disorders and in contributing to the understanding of their pathobiology. I want to pursue a career in research and teaching since I enjoy teaching genetics to students and investigating the important and unsolved research questions.
